# The Atrial Fibrillation Health Literacy Information Technology System: Pilot Assessment

**DOI:** 10.2196/cardio.8543

**Published:** 2017-12-12

**Authors:** Jared W Magnani, Courtney L Schlusser, Everlyne Kimani, Bruce L Rollman, Michael K Paasche-Orlow, Timothy W Bickmore

**Affiliations:** ^1^ Division of Cardiology, Department of Medicine, UPMC Heart and Vascular Institute University of Pittsburgh Pittsburgh, PA United States; ^2^ Center for Behavioral Health Smart Technology Department of Medicine University of Pittsburgh Pittsburgh, PA United States; ^3^ College of Computer and Information Science Northeastern University Boston, MA United States; ^4^ Section of General Internal Medicine Department of Medicine Boston University Boston, MA United States

**Keywords:** atrial fibrillation, mHealth, health-related quality of life, medication adherence

## Abstract

**Background:**

Atrial fibrillation (AF) is a highly prevalent heart rhythm condition that has significant associated morbidity and requires chronic treatment. Mobile health (mHealth) technologies have the potential to enhance multiple aspects of AF care, including education, monitoring of symptoms, and encouraging and tracking medication adherence. We have previously implemented and tested relational agents to improve outcomes in chronic disease and sought to develop a smartphone-based relational agent for improving patient-centered outcomes in AF.

**Objective:**

The objective of this study was to pilot a smartphone-based relational agent as preparation for a randomized clinical trial, the Atrial Fibrillation Health Literacy Information Technology Trial (AF-LITT).

**Methods:**

We developed the relational agent for use by a smartphone consistent with our prior approaches. We programmed the relational agent as a computer-animated agent to simulate a face-to-face conversation and to serve as a health counselor or coach specific to AF. Relational agent’s dialogue content, informed by a review of literature, focused on patient-centered domains and qualitative interviews with patients with AF, encompassed AF education, common symptoms, adherence challenges, and patient activation. We established that the content was accessible to individuals with limited health or computer literacy. Relational agent content coordinated with use of the smartphone AliveCor Kardia heart rate and rhythm monitor. Participants (N=31) were recruited as a convenience cohort from ambulatory clinical sites and instructed to use the relational agent and Kardia for 30 days. We collected demographic, social, and clinical characteristics and conducted baseline and 30-day assessments of health-related quality of life (HRQoL) with the Atrial Fibrillation Effect on Quality of life (AFEQT) measure; self-reported medication adherence with the Morisky 8-item Medication Adherence Scale (MMAS-8); and patient activation with the Patient Activation Measure (PAM).

**Results:**

Participants (mean age 68 [SD 11]; 39% [12/31] women) used the relational agent for an average 17.8 (SD 10.0) days. The mean number of independent log-ins was 19.6 (SD 10.7), with a median of 20 times over 30 days. The mean number of Kardia uses was 26.5 (SD 5.9), and participants using Kardia were in AF for 14.3 (SD 11.0) days. AFEQT scores improved significantly from 64.5 (SD 22.9) at baseline to 76.3 (SD 19.4) units at 30 days (*P*<.01). We observed marginal but statistically significant improvement in self-reported medication adherence (baseline: 7.3 [SD 0.9], 30 days: 7.7 [SD 0.5]; *P*=.01). Assessments of acceptability identified that most of the participants found the relational agent useful, informative, and trustworthy.

**Conclusions:**

We piloted a 30-day smartphone-based intervention that combined a relational agent with dedicated content for AF alongside Kardia heart rate and rhythm monitoring. Pilot participants had favorable improvements in HRQoL and self-reported medication adherence, as well as positive responses to the intervention. These data will guide a larger, enhanced randomized trial implementing the smartphone relational agent and the Kardia monitor system.

## Introduction

Atrial fibrillation (AF) is a chronic and highly prevalent heart rhythm condition [1]. Treatment for AF requires long-term adherence to complex therapies that include anticoagulants and medications for heart rate and rhythm control [2]. AF has variable symptoms that fluctuate in severity and is associated with increased risks of adverse outcomes such as heart failure, stroke, hospitalization, and mortality [3]. The diverse treatments, symptoms, and potential for adverse outcomes combine to make AF a challenging condition for patients, which is associated with poor health-related quality of life (HRQoL) [4-6]. AF professional guidelines prioritize routine symptom assessment and monitoring to guide the treatment course [2]. Patients may have varying access to care, which may be further compromised by challenges to health literacy and obstacles to medication adherence [7]. Similarly, intermittent visits are the standard for clinical care, and patients may experience problems related to this chronic, complex disease between such visits.

**Figure 1:**
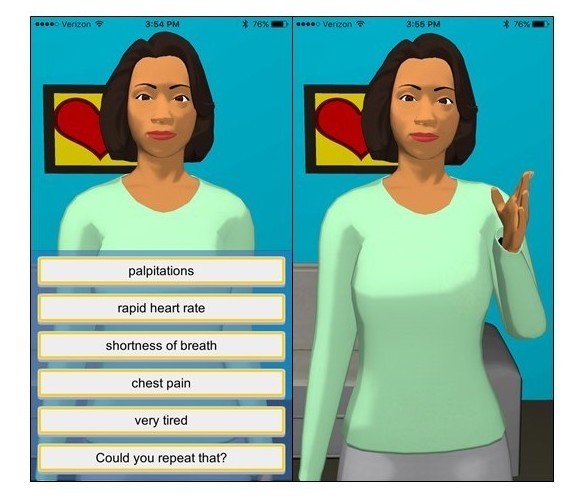
Screenshots of the relational agent: (a) symptoms menu and (b) an emphatic gesture.

Mobile health (mHealth) technologies have the potential to enhance multiple aspects of patient care by providing education, monitoring, of symptoms, and encouraging and tracking adherence to long-term pharmacological therapies or behaviors. We have developed and used relational agents in prior contexts to augment patient-centered health care. The relational agent is an animated character that provides health education, monitoring, and problem solving with speech, facial expression, and body gestures. Users converse and respond with on-screen selections, which in turn activate further dialogue with the agent. Figure 1 shows screenshots of the relational eliciting symptoms pertinent to AF and gesturing to enhance educational content. We propose the relational agent as a vehicle for improving chronic disease self-management, which provides practical strategies to negotiate the long-term self-care and medication adherence that are essential for patient’s success with a chronic disease such as AF [8].

Our prior work has demonstrated that a relational agent is an effective health educator for all levels of health literacy and improves self-management and HRQoL [9-13]. In randomized studies, we have demonstrated that the use of the relational agent improves pharmacological adherence, diet, and physical activity, and it reduces the likelihood of hospital readmission [14-16]. Specific examples of our application of the relational agent to improve adherence include a clinical trial with 263 older adults to increase adherence over 12 months of routine physical activity [17] and the promotion of adherence to antipsychotic medication in schizophrenia (n=20) [18]. In an additional larger application, we examined the effect of the relational agent to improve communication with patients at hospital discharge (N=764) and found that the agent was associated with reduced rates of readmission and improved patient satisfaction [19]. The relational agent is a multifaceted intervention. It serves as a nonjudgmental empathetic coach and educator to provide patient-centered guidance.

To our knowledge, relational agents have had limited application in cardiovascular disease and have not been used in AF previously. Current ambulatory monitoring in AF focuses predominantly on AF detection [20-22]. Although such efforts have a clinical application, they may not provide patient-centered health coaching. The relational agent, in contrast, can assist patients to develop essential skills to understand monitoring results and respond to them.

Relational agents may be combined with other mHealth technology to augment their health-related impact. The AliveCor Kardia mobile heart rhythm monitor (AliveCor, Inc, Mountain View, CA) or Kardia for simplicity, allows individuals to monitor heart rate and rhythm with the help of a smartphone with results being uploaded for centralized review. Kardia has been used prospectively in various studies, again principally for AF detection [23-25]. We consider that Kardia may be combined with the relational agent described here to use the two synergistically. Kardia may facilitate correlation of symptoms reported to the relational agent, and relational agent content may reciprocally encourage individuals to take Kardia recordings. For example, patients may report symptoms to the relational agent, which in turn could prompt relational agent content that encourages Kardia use, and both applications may corroborate and support the documentation provided by the other. As the use of the relational agent and Kardia are time-stamped, the results may be incorporated as data to guide and direct clinical management.

We developed a relational agent for AF that integrated with Kardia. We sought to pilot this mHealth intervention and then use the results to guide the development of the clinical trial, the Atrial Fibrillation Health Literacy Information Technology Trial (AF-LITT), registered at ClinicalTrials.gov (NCT03093558). We report here on the initial pilot (n=31) of this mHealth intervention for preliminary results to guide and enhance the randomized controlled trial (RCT).

## Methods

### Relational Agent Development and Content

The intervention includes a computer-animated relational agent that simulates a face-to-face conversation with a health counselor or coach using synthetic speech that accompanies animated conversational behavior (Figure 1) [10,13,16,26,27]. Our prior work has demonstrated that the relational agent as a vehicle for human-computer interaction is accessible to individuals with limited computer literacy and to older adults [11,26,27]. Our work has further shown that the relational agent fosters a therapeutic alliance with patients of diverse socioeconomic backgrounds and health literacy [28-30].

Users make selections via the smartphone’s touch screen, and this selection directs further interaction and content provided by the relational agent. Communication with users includes nonverbal conversational behaviors, such as facial expressions for empathy or hand gestures for emphasis, which reinforce communication and facilitate empathetic expression. The content may be tailored for individual use, such that the relational agent addresses users by name and appropriate time context (eg, Good morning). In addition, the relational agent may be programmed to refer to prior content areas to obtain repeated assessments and follow the resolution of reported problems. We provided the agent with a name to facilitate user interaction, which is consistent with our other relational agent interventions; in this case, we designated the agent as “Tanya”.

The relational agent content was developed by review of patient-centered domains, review of the literature, and qualitative interviews with patients with AF. In brief, we had a focus group consisting of patients with AF and receiving chronic anticoagulation. We asked patient participants open-ended questions about their experience of AF and the challenges and gaps that they experienced in their care. Our work is consistent with others in that the patients have a poor understanding of the condition, experience myriad symptoms, and find adherence challenging [31]. We consequently used these domains to develop the pilot of the relational agent, listed in Table 1.

**Table 1:** Summary of relational agent domain content.

Domain	Module content
Education	Causes of AF^a^
	AF treatment strategies
	Stroke prevention in AF
	AliveCor Kardia use, troubleshooting
Symptoms	Overview of common symptoms
	Chest pain and chest pressure
	Heart racing or palpitations
	Dyspnea and shortness of breath
	Fatigue
Adherence	Overview of adherence
	Adherence to medications
	Adherence barriers
	Strategies to address barriers
Patient activation	Goals for self-management
	Preparing for the medical encounter

^a^AF: atrial fibrillation.

Education spanned the causes of AF and its associated risk factors, treatments, and adverse events. Symptoms consisted of assessment of frequency and severity of shortness of breath, chest pain or discomfort, fatigue, and palpitations or the sensation of a racing or irregular heartbeat. Adherence content focused on common challenges to medication adherence, such as forgetfulness, affordability, access to medications, transportation, and the patient-physician relationship, along with general strategies to address these obstacles. Activation content entailed articulating goals of care and preparing for the medical encounter. For the concomitant use of Kardia, users were prompted to use Kardia daily and when they experienced symptoms.

### Relational Agent and Kardia Use Ascertainment

The relational agent application and the Kardia monitor transmitted their data to a central server for monitoring, data collection, and analysis. Relational agent data included the date and time of use, duration, and specific domains of content accessed. Kardia data included the date of recording and recording results with a classification of heart rhythm (AF, sinus, or other) and heart rates in AF.

### Cohort Participants

Participants were recruited from a convenience cohort of patients with prevalent AF, who were receiving care at ambulatory facilities of the University of Pittsburgh Medical Center in Pittsburgh, PA. Inclusion criteria included adult (age ≥18), a diagnosis of nonvalvular AF as ascertained by review of the electronic health record, CHA_2_ DS_2_-VASc score ≥2 [32], and receiving oral anticoagulation. Participants were excluded for having an identified extracardiac cause of AF (such as sepsis or thyroid disease), as the management of AF in such context may differ based upon the underlying etiology [2]; inability to provide accurate three-word recall; inability to provide informed consent; or being non-English speaking. Individuals were provided with an iPhone 6 (Apple, Cupertino, CA) for the duration of the study and instructions on the use of the relational agent and Kardia system. The study was approved by the University of Pittsburgh School of Medicine’s Institutional Review Board.

### Participant Assessments

Age, race, smoking status, highest level of education (less than high school, high school graduate, or vocational or trade school; some college with no degree or associate degree; bachelor degree; or graduate or postcollege professional school), and annual income (up to US $19,000; US $20-34,999; US $35-49,999; US $50-74,999; and ≥US $100,000) were obtained by participant self-report. Date of diagnosis with AF was obtained by self-report and the medical record. Body mass index (BMI) was calculated from weight (kg) divided by height (meters squared). Smoking status (current or prior smoking) and pack-years were obtained by self-report. Clinical covariates were derived from medical record problem lists and included hypertension, diabetes, cardiovascular disease, heart failure, and prior stroke or transient ischemic attack. Medications (anticoagulants, antiarrhythmics, and rate control agents) and treatment for AF (history of pharmacological or electrical cardioversion, pulmonary vein isolation) were obtained by self-report with corroboration from the medical record. Health literacy was measured by the Short-Test of Functional Health Literacy in Adults (S-TOFHLA) [33], which was selected for its broad use as a measure for health literacy assessment, scored from 1 to 36, with a score of ≤23 indicating limited health literacy.

The Atrial Fibrillation Effect on Quality of life (AFEQT) [34], the Morisky 8-item Medication Adherence Scale (MMAS-8) [35], and the Patient Activation Measure (PAM) [36] were administered to participants at baseline and at study completion, that is, day 30. HRQoL was ascertained with the AFEQT questionnaire, a validated instrument specific to AF-related HRQoL. The AFEQT consists of a 20-item instrument that measures the effects of AF on HRQoL in the domains of symptoms, daily activities, treatment concerns, and treatment satisfaction, in addition to providing a global measure. Scores range from 0 to 100, with higher scores indicating superior HRQoL. Medication adherence was determined by self-report with the MMAS-8, which has been validated in diverse clinical conditions and is additionally used in studies of participants with limited health literacy [37,38]. The MMAS-8 ranges from 0 to 8, with higher scores indicating greater medication adherence. The 13-item PAM was used to ascertain patient activation, which has been conceptualized as the confidence, skills, and knowledge for patient self-management. PAM scores are tabulated from 0 to 100 and then converted from level 0 to level 5 as described elsewhere [39]. We elected not to include an assessment of AF patient knowledge, as the evidence that patient knowledge improves medication adherence in AF is scant [40].

### Coaching, Acceptability, and Remuneration

Participants were contacted by telephone at their primary phone number on days 7, 14, and 21 to discuss problems with using the relational agent or Kardia. Individuals without relational agent use for ≥3 days were additionally contacted for support and troubleshooting. Qualitative interviews were conducted with participants at 30 days. Participants completed a 22-item instrument that evaluated their experience with the relational agent using 7-point Likert scale items. Pilot study participants received US $20 for the initial and final visit, and they were provided with a payment of US $1/day each for the use of the relational agent and Kardia, as an incentive for use and continued adherence. Therefore, the participants were able to receive a maximum of US $100 for two study visits and 30-day use of both instruments.

### Statistical Analysis

We summarized the distributions of categorical and continuous variables. We used paired, 2-tailed *t* tests to examine the differences at baseline and 30 days in HRQoL with the AFEQT, self-reported adherence with the MMAS-8, and patient activation with the PAM. A *P* value of <.05 was considered statistically significant.

## Results

The pilot cohort enrolled 31 participants with a mean age of 68 (SD 11) years, 39% of the participants were women, and predominantly belonging to the white race (94%, 29/31). Further characteristics of the cohort, which are summarized in Table 2, included both limited education (not having completed college, 68%, 21/31) and annual income (<US $35,000, 39%, 12/31; with the majority reporting <US $19,000). Obesity was common, with a mean BMI of 30.8 (SD 8.1), and multiple participants had diabetes, cardiovascular disease, a history of heart failure, or prior stroke or transient ischemic attack.

**Table 2:** Descriptive characteristics of the pilot cohort (N=31).

Characteristics		Cohort (N=31)
Age in years, mean (SD)	68 (11)	
Women, n (%)	12 (39)	
White race, n (%)	29 (94)	
Education, <College, n (%)	21 (68)	
Annual income, <US $35,000, n (%)	12 (39)	
S-TOFHLA^a^≤23, n (%)	7 (23)	
BMI^b^, kg/m, mean (SD)	30.8 (8.8)	
Current smoker, n (%)	5 (16)	
Hypertension, n (%)	21 (68)	
Diabetes, n (%)	4 (13)	
Prevalent CVD^c^, n (%)	4 (13)	
Prevalent heart failure, n (%)	7 (23)	
Prior stroke or TIA^d^, n (%)	5 (16)	

^a^S-TOFHLA: Short-Test of Functional Health Literacy in Adults.

^b^BMI: body mass index.

^c^CVD: cardiovascular disease.

^d^TIA: transient ischemic attack.

**Table 3:** Selected assessments of the pilot cohort at baseline and 30 days.

Assessments		Baseline	30 days	*P*
AFEQT^a^, global score		64.5 (22.9)	76.3 (19.4)	<.01
**AFEQT domains**				
	Symptoms	74.6 (24.1)	80.7 (21.4)	.07
	Daily activities	56.0 (27.8)	65.2 (26.1)	.01
	Treatment concerns	66.7 (26.2)	74.6 (22.6)	.08
	Treatment satisfaction	71.2 (25.4)	72.9 (27.6)	.71
MMAS^b^		7.3 (0.9)	7.7 (0.5)	.01
PAM^c^		3.0 (0.8)	3.4 (0.7)	.33

^a^AFEQT: Atrial Fibrillation Effect on Quality of life.

^b^MMAS: Morisky 8-item Medication Adherence Scale.

^c^PAM: Patient Activation Measure.

Study participants used the relational agent for a range extending from 3 to 30 days, with a median of 20 days’ usage that averaged 17.8 (SD 10.0) days of use. The number of log-ins to the relational agent ranged from 4 to 43, with a median of 20 and average of 19.6 (SD 10.7) uses. The median number of Kardia uses was 29, extending from 5 to 30, with a mean of 26.5 (SD 5.9) uses. Participants were in AF, a median of 13 days during the 30-day study period.

The baseline and 30-day assessments of AFEQT, self-reported adherence, and PAM scores are summarized in Table 3. The global AFEQT score was significantly improved from baseline to day 30, as was the daily activity component of the AFEQT. The domains of symptoms and treatment concerns had improvement from baseline to day 30 that was not statistically significant. The AFEQT domain of treatment satisfaction remained unchanged from baseline to day 30. Self-reported adherence with the MMAS-8 was marginally but significantly improved. The improvement in patient activation as measured by the PAM did not reach statistical significance.

**Table 4:** Relational agent acceptability assessment using a Likert-score ranging from 1 to 7.

Item	Description of range	Median (range)
How satisfied were you with Tanya?	1=Not at all; 7=Very satisfied	5 (1-7)
How easy was talking to Tanya?	1=Easy; 7=Difficult	1 (1-7)
How much would you like to continue working with Tanya?	1=Not at all; 7=Very much	5 (1-7)
How much do you like Tanya?	1=Not at all; 7=Very much	5 (1-7)
How would you characterize your relationship with Tanya?	1=Complete stranger; 7=Close friend	4 (1-7)
How much do you trust Tanya?	1=Not at all; 7=Very much	5 (3-7)
How much do you feel that Tanya cares about you?	1=Not at all; 7=Very much	5 (3-7)
How much do you feel that you and Tanya understand each other?	1=Not at all; 7=Very much	5 (1-7)
I feel uncomfortable with Tanya.	1=Disagree completely; 7=Agree completely	1 (1-5)
Tanya and I understand each other.	1=Disagree completely; 7=Agree completely	4 (1-7)
I believe Tanya likes me.	1=Disagree completely; 7=Agree completely	4 (1-7)
I believe Tanya is genuinely concerned about my welfare.	1=Disagree completely; 7=Agree completely	5 (1-7)
Tanya and I respect each other.	1=Disagree completely; 7=Agree completely	5 (1-7)
I feel that Tanya is not totally honest about her feelings toward me.	1=Disagree completely; 7=Agree completely	1 (1-5)
I am confident in Tanya’s ability to help me.	1=Disagree completely; 7=Agree completely	5 (3-7)
I feel that Tanya appreciates me.	1=Disagree completely; 7=Agree completely	4 (1-7)
Tanya and I trust one another.	1=Disagree completely; 7=Agree completely	4 (1-7)
My relationship with Tanya is very important to me.	1=Disagree completely; 7=Agree completely	4 (1-7)
I have the feeling that if I say or do the wrong things, Tanya will stop working with me.	1=Disagree completely; 7=Agree completely	4 (1-7)
I feel Tanya cares about me even when I do things she does not approve of.	1=Disagree completely; 7=Agree completely	1 (1-4)

Our postuse assessments of the relational agent showed moderately strong acceptability of the agent. Thirteen of 31 participants reported either 6 or 7 on a 7-point Likert scale (1=Not at all; 7=Very satisfied) concerning their satisfaction with Tanya. Twenty-three participants selected 1 on the 7-point Likert scale, indicating that talking with Tanya was easy (1=Easy; 7=Difficult). When asked about continuing to work with Tanya, 17 indicated a strong willingness to continue, 9 would consider continuing with additional modules, whereas 5 indicated no interest. When scoring how much participants liked Tanya, 21 indicated a 5 or higher on the 7-point Likert scale (1=Not at all; 7=Very much). Participants further endorsed comfort with the agent and a belief that the agent is genuinely concerned about their welfare. These results and the remaining assessments of participants’ experience with the relational agent are summarized in Table 4.

## Discussion

### Principal Findings

We piloted a 30-day intervention that combined a smartphone-based relational agent and the AliveCor Kardia heart rhythm monitor. Our chief findings were that the participants in this small-sized pilot reported improved HRQoL specific to AF and self-reported medication adherence. We tracked use of the relational agent and Kardia and identified that participants used them reliably over the 30-day intervention period. Finally, we demonstrated that the relational agent developed for this intervention had moderate to high acceptability by study participants.

There is a growing interest in mHealth to enhance patient engagement and improve patient outcomes. Our work focuses on implementing a relational agent, termed here the relational agent, which has distinct advantages as a mHealth intervention. The agent uses conversational behaviors—empathic tone, hand gestures, and facial expressions—to convey information. The responses to our postsurvey acceptability assessment suggest that users find the agent accessible, informative, and trustworthy. These results are similar to our prior work with relational agents, where we have used the relational agent as a nonjudgmental, empathetic coach to offer patient-centered guidance [15,41,42]. Relational agents provide an effective medium for health communication and counseling, especially for patients with limited health literacy. Health literacy is associated with poor self-management in multiple diverse conditions [11,43-45]. In our prior work, we have used the relational agent to improve health outcomes in individuals with limited health literacy [11,26,27] and have developed the content of our relational agent for AF to be accessible for such individuals. Health literacy has relevance to mHealth development, accessibility, and successful implementation; mHealth focused toward health literacy has the potential to reduce disparities in vulnerable populations, where limited health literacy is more prevalent [27].

In addition, the relational agent has the potential to assist users in real time. Content may be programmed to address immediate problems, suggest solutions, and then track their resolution. The relational agent content used in this pilot focused on major symptoms and then inquired about their status. Such content and the monitoring of responses may be implemented in conjunction with the Kardia. Thus, it is feasible to track symptoms while assessing their correlation with simultaneous heart rhythm or rate monitoring. In our next iteration, we expect to use symptoms and Kardia rate and rhythm monitoring to guide interventions in real time. We expect in our RCT to follow participants prospectively and longitudinally using a dashboard; so, we can respond to assist clinicians with enhanced management strategies.

The relational agent can promote self-management that is central to success with a chronic disease. The self-management model recognizes chronic disease management as a long-term endeavor with an uncertain prognosis and varied course [46]. AF is well suited for the self-management model because of its complex treatments, the potential for myriad complications, and necessity of patient adherence and engagement. We have developed our relational agent content guided by the self-management model because of the focus on sustained self-care and prevention in a chronic disease such as AF. The relational agent can promote patient education essential to self-management [47]. mHealth interventions that promote the self-management model must have the capacity to address the long-term aspects of chronic disease management. The relational agent is well suited, given its accessibility by a smartphone and capacity for expanded content delivered by the agent.

The adverse impact of AF on HRQoL has been well established [48-50]. The strengths of the AFEQT include its validation and development as a measure specific to AF [34,51]. A systematic review concluded that the AFEQT has stronger construct validity than other HRQoL measures specific to AF [52]. HRQoL has received increased emphasis as a patient-reported outcome, but it remains absent from many cardiovascular trials, including those focused on AF [53]. Although we observed a significant difference in baseline and 30-day AFEQT scores, the change did not meet the 19-unit, minimal important difference in the instrument that has previously been identified [51]. However, that threshold for a minimal important difference was ascertained over 3 months in a much larger cohort (n=210). In addition, we observed significant changes in the global AFEQT score and its domain of daily activities, and a trend toward improvement in symptoms and treatment concerns that did not reach our threshold for statistical significance. We did not observe improvement in treatment satisfaction, possibly because our 30-day intervention was too limited in duration for participants’ treatment to undergo modification. We intend to conduct a more sustained intervention, which will provide confirmation of the efficacy of the relational agent to improve HRQoL. Real-time monitoring with targeted interventions may also improve treatment satisfaction.

Medication adherence is a prominent goal for chronic disease management and crucial in AF. In addition to increased medical costs and hospitalization risk, AF is associated with adverse events such as strokes and heart failure. Strokes may be prevented with regular use of anticoagulation, either vitamin K antagonist, such as warfarin, or one of the newer oral anticoagulants that do not require routine monitoring. However, poor adherence to anticoagulation in AF is common. Discontinuation rates in clinical trials for the novel oral anticoagulants (NOAC) were 15% to 34% [54-56]. Claims data indicate that only 49% to 67% of NOAC users have satisfactory fill rates 9 to 12 months following the initial prescription [57,58]. For warfarin users, annual discontinuation reaches 27% to 33% [59]. Clinical trials and registries further affirm high discontinuation rates for both warfarin and NOAC users [57,60]. As AF is a lifelong condition, deescalation of anticoagulation is only justified if continued treatment is contraindicated, and long-term adherence is essential for stroke prevention. We expect to continue to refine and develop the relational agent used in this application. Our goals include enhanced adherence content for active problem solving of adherence challenges and obstacles; determining the reliability of the relational agent to measure adherence, compared with a gold standard such as medication event monitoring systems; and measuring the efficacy of the relational agent to improve adherence using objective measures rather than relying on self-reporting, which is subject to multiple biases [61].

### Limitations

We also consider that our pilot study reported here has important limitations. First, the study sample was small (n=31), selected as a convenience cohort, and without randomization. We recognize a biased selection approach that may likely influence our results. For example, individuals recruited for this pilot may be more enthusiastic about the use of new technology, and they, consequently, demonstrate a greater likelihood of daily use than a more generalizable cohort. Second, individuals received repeated assessments using the same instruments over the 30-day study period. It is possible that the repeat measurement may modify or influence participant’s self-report of HRQoL or medication adherence. Third, participants were contacted during the study and offered support using the technology. Participants’ use of the intervention may have been influenced by such contact. However, for mHealth to be successful, it must be accessible to users. Fourth, our limited pilot cohort is racially homogeneous, as only 2 participants belonged to the nonwhite race. Enhanced recruitment of ethnic and racial minorities—those most likely to experience more severe differences in AF outcomes [62]—is critical to address disparities in AF. Fifth, we recruited from a limited number of ambulatory sites without control for practice patterns or clinical approaches. It is possible that there is residual confounding due to how clinicians caring for such a small-sized cohort may approach AF. Sixth, we had variable adherence to the intervention in this pilot. We are using these results to refine and improve relational agent content. Finally, the statistical assessments used to compare the baseline and 30-day measures were not adjusted for age, sex, and duration of AF, health literacy or other covariates. Such factors may have influenced adherence to the relational agent and consistency of use. Given our small sample size, stratified analyses would neither be statistically efficient nor meaningful. Furthermore, identifying which components of the multifaceted intervention promoted use would be challenging in this sample size. In subsequent, larger studies, we intend to measure the effects of the relational agent on prespecified subgroups.

### Conclusions

In conclusion, we present results on the pilot use of a smartphone-based relational agent in combination with heart rate and rhythm monitoring. In our limited-sized pilot study conducted over 30 days, we identified strong adherence to the intervention and showed significant improvements in HRQoL and self-reported adherence. Participants found the relational agent acceptable. These results will guide the development and implementation of a more comprehensive relational agent for guiding long-term AF management to improve patient self-management and the experience of a highly morbid condition.

## Abbreviations

AF: atrial fibrillation
AF-LITT: Atrial Fibrillation Health Literacy Information Technology Trial
AFEQT: Atrial Fibrillation Effect on Quality of life
HRQoL: health-related quality of life
mHealth: mobile health
MMAS-8: Morisky 8-item Medication Adherence Scale
NOAC: novel oral anticoagulants
PAM: Patient Activation Measure
S-TOFHLA: Short-Test of Functional Health Literacy in Adults
